# The Role of Inflammatory and Anti-Inflammatory Cytokines in the Pathogenesis of Osteoarthritis

**DOI:** 10.1155/2014/561459

**Published:** 2014-04-30

**Authors:** Piotr Wojdasiewicz, Łukasz A. Poniatowski, Dariusz Szukiewicz

**Affiliations:** Department of General and Experimental Pathology, Second Faculty of Medicine, Medical University of Warsaw, Pawińskiego 3c, 02-106 Warsaw, Poland

## Abstract

Osteoarthritis (OA) is the most common chronic disease of human joints. The basis of pathologic changes involves all the tissues forming the joint; already, at an early stage, it has the nature of inflammation with varying degrees of severity. An analysis of the complex relationships indicates that the processes taking place inside the joint are not merely a set that (seemingly) only includes catabolic effects. Apart from them, anti-inflammatory anabolic processes also occur continually. These phenomena are driven by various mediators, of which the key role is attributed to the interactions within the cytokine network. The most important group controlling the disease seems to be inflammatory cytokines, including IL-1**β**, TNF**α**, IL-6, IL-15, IL-17, and IL-18. The second group with antagonistic effect is formed by cytokines known as anti-inflammatory cytokines such as IL-4, IL-10, and IL-13. The role of inflammatory and anti-inflammatory cytokines in the pathogenesis of OA with respect to inter- and intracellular signaling pathways is still under investigation. This paper summarizes the current state of knowledge. The cytokine network in OA is put in the context of cells involved in this degenerative joint disease. The possibilities for further implementation of new therapeutic strategies in OA are also pointed.

## 1. Introduction

**Figure 1:**
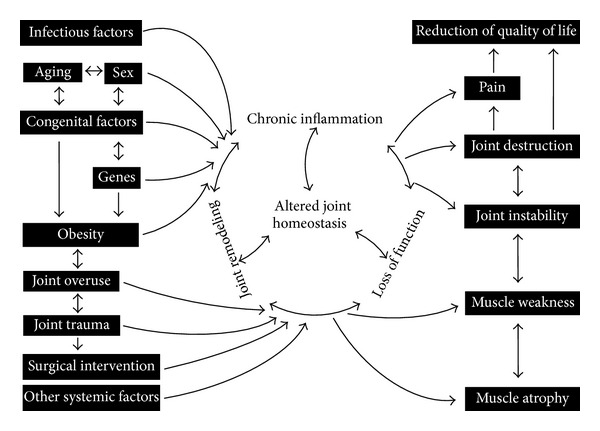
Schematic of a closed disease circle comprising the disease progression of osteoarthritis taking into account its causes and consequences.

The most common chronic and currently regarded as potentially irreversible disease that affects the joints is osteoarthritis (OA) [1, 2]. The constantly growing number of causes for the development of the disease includes, for example, genetic predisposition, aging, obesity, trauma, and other systemic diseases (Figure 1). Although we are dealing with a diverse aetiology, which is most often the result of a number of overlapping factors, processes occur during the development of the pathomechanisms of disease at the tissue, cellular and ultrastructural level that gradually take a similar nature, resulting in the phenotypic image of OA. According to the latest medical knowledge, the participation of the immune system in the development and progression of OA is one of the key elements in the pathogenesis of the disease [3]. Currently, many independent authors are focused on identifying and describing the factors responsible for the development of inflammatory processes involved in OA [4]. An analysis of the ever-increasing number of reports directs attention to the special role of the cytokine network in the pathogenesis of OA. During the progression of OA, the production and operation of various cytokines can vary depending on the duration and severity of the disease [5]. The most important effect that cytokines have involves disturbing the catabolism and anabolism processes, particularly important in tissues that are often subject to high mechanical load, such as human joints [6]. As a result of disrupting the said balance, there is a progressive degeneration of articular cartilage performing a key role in the biomechanics of each joint and other components of the joint, which results in the development of a difficult-to-interrupt disease process that involves both inflammatory, degradation, and production processes, which together lead to a gradual loss of joint function and pain. Due to the effect of cytokines in the context of an inflammatory disease such as OA, they can be divided into inflammatory and anti-inflammatory [7]. It should be noted that the pathophysiological processes occurring in the joint affected by OA are largely mediated by inflammatory cytokines and other mediators intensifying catabolic effects. At the same time, one should not underestimate the role of anti-inflammatory cytokines that may modulate an inflammatory response and act protectively on joint tissue.

Although we are not able to prevent some of the causes of OA (genetic factors, aging, and trauma), we can try to alter the course of disease at the level of cellular communication through a better understanding of the sensitive dependences in the network of cytokines. This opens the possibility of using suitable targeted treatment with the use of antibodies and other recombinant biological factors that interrupt the self-perpetuating processes of the closed illness process [8]. This review aims to provide accurate and current knowledge on the role of inflammatory and anti-inflammatory cytokines in the pathogenesis of OA. It is worth noting that the role of anti-inflammatory cytokines in the context of OA has not been studied comprehensively so far.

## 2. Inflammatory Cytokines

The group of inflammatory cytokines is the most important group of compounds participating in the pathogenesis of OA. Their participation is by far the most widely and accurately documented in literature. They are responsible to the greatest extent for the loss of metabolic homeostasis of tissues forming joints by promoting catabolic and destructive processes. The key role they play in the pathogenesis of OA is a result of the effect these compounds have on the majority of cells that are in the joint and the impact via intracellular pathways of signal transduction on the production of cytokines as well as other inflammatory compounds and enzymes. Among the many representatives of this group, the greatest importance is attributed to IL-1*β*, TNF*α*, IL-6, IL-15, IL-17, and IL-18.

### 2.1. Interleukin-1 Beta (IL-1*β*)

**Figure 2:**
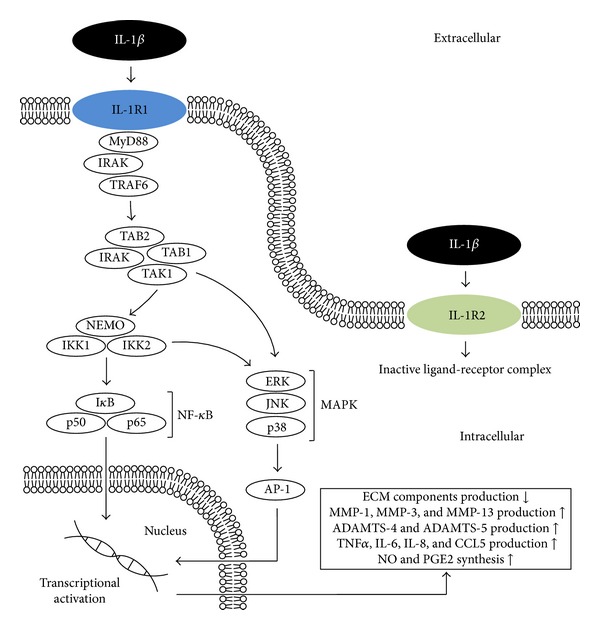
IL-1*β* associated intracellular signaling pathways and downstream cellular targets and effects. IL-1R1: interleukin-1 receptor, type 1; IL-1R2: interleukin-1 receptor, type 2; MyD88: myeloid differentiation primary response gene (88); IRAK: interleukin-1 receptor-associated kinase; TRAF6: TNF receptor-associated factor 6; TAK1: also known as mitogen-activated protein kinase kinase kinase 7 (MAP3K7); TAB1: also known as mitogen-activated protein kinase kinase kinase 7 interacting protein 1 (MAP3K7IP1); TAB2: also known as mitogen-activated protein kinase kinase kinase 7 interacting protein 2 (MAP3K7IP2); p50, p65: subunits of proteins forming NF-*κ*B; I*κ*B: (inhibitor of *κ*B) an endogenous complex of proteins inhibiting the activation of NF-*κ*B; IKK1,2/NEMO: NF-*κ*B inhibitor kinase 1,2 (I*κ*B kinase 1,2)/NF-*κ*B kinase inhibitor (NF-*κ*B essential modulator); ERK: extracellular-signal-regulated kinase; JNK: c-Jun N-terminal kinase; p38: p38 mitogen-activated protein kinases; MAPK: mitogen-activated protein kinases; AP-1: activator protein 1.

Interleukin-1 beta (IL-1*β*) is considered one of the key cytokines involved in the pathogenesis of OA; it induces inflammatory reactions and catabolic effect independently as well as being combined with other mediators with respect to the articular cartilage and other elements of joints. It is one of the 11 representatives of the IL-1 family (IL-1F) [9] (Figure 2). It is originally formed as a cytosolic precursor protein (pro-IL-1*β*) consisting of 269 amino acid residues [9, 10]. The preparation of the active form of IL-1*β* with 153 amino acid residues is the result of intracellular proteolysis carried out by the enzyme Caspase 1 (IL-1*β* converting enzyme, ICE), followed by the release into the extracellular space [11]. Its synthesis in the joint is governed by chondrocytes, osteoblasts, cells forming the synovial membrane, and mononuclear cells that were previously present in the joint or infiltrated its structure during the inflammatory response [4, 12–16]. Patients with OA have an elevated level of IL-1*β* in both the synovial fluid, synovial membrane, cartilage, and the subchondral bone layer [12, 13, 15, 17]. The biological activation of cells by IL-1*β* is mediated by interaction with the membrane receptor, namely, the IL-1R1 (IL-1RI, CD121a), which can also bind IL-1*α* and IL-1Ra [18]. Another receptor capable of binding IL-1*β* is IL-1R2 (IL-1RII, CD121b), which after binding a ligand, such as the IL-1*α*, IL-1*β*, or IL-1Ra, forms an inactive ligand-receptor complex, showing no ability to communicate and activate the intracellular signal [19]. At this point it is worth mentioning the presence of the receptor antagonist IL-1Ra produced by certain cells of the joint, which (as noted above) can bond with IL-1R1 and IL-1R2, thereby blocking their connection with IL-1*β*, which may to some extent affect the decrease in the activity of IL-1*β* [20, 21]. The expression of the IL-1R1 receptor is increased in patients with OA on the surface of chondrocytes and fibroblast-like synoviocytes (FLS) compared to treatment groups [22, 23]. Binding the IL-1*β* to a receptor of the TLR family, such as IL-1R1, is followed by recruitment of additional IL-1R3 chain (IL-1RAcP), thereby forming a complex which, through its intracellular domain Toll-IL-1R (TIR), recruits the adapter protein MyD88 [24]. The entire previously described complex binds serine-threonine kinases of the IRAK group, which affect the TRAF6 protein, which induces further binding of TAK1, TAB1, and TAB2 [25]. TAK1 affects the phosphorylation of the I*κ*B kinase complex (IKK), thereby activating the transcription factor NF-*κ*B [25]. Moreover, p38MAPK and c-Jun N-terminal kinase (JNK) are also activated [24, 25]. Activation of these transcription factors very quickly results in the expression of hundreds of genes whose products include other cytokines, chemokines, adhesion molecules, inflammatory mediators, and enzymes [26]. The effect of IL-1*β* is manifested by its significant effect on the metabolism of cells and the extracellular matrix (ECM) [27]. In the course of the disease, the gradual loss of articular cartilage is of paramount importance. Many studies confirm that the effect of IL-1*β* blocks chondrocytes in the context of the synthesis of ECM components, interfering with the synthesis of the key structural proteins such as type-II collagen and aggrecan [28, 29]. In addition to the decrease in the synthesis of the building blocks, the IL-1*β* affects the operation of chondrocytes in the synthesis of enzymes from the group of metalloproteinases (MMPs), mainly interstitial collagenase (MMP-1), stromelysin-1 (MMP-3), and collagenase 3 (MMP-13), which have a destructive effect on cartilage components [30–32]. Besides the induction of enzymes of the MMPs family, IL-1*β* affects the chondrocytes' production of ADAMTS metalloproteinases, which are responsible for the proteolysis of aggrecan molecules [33]. A major role is attributed to ADAMTS-4, whose production is stimulated by both IL-1*β* and TNF*α*, while ADAMTS-5 has no correlation with the influence of cytokines and is produced constitutively [33, 34]. Chondrocytes subjected to the effect of IL-1*β* and TNF*α* also tend to age more rapidly and to induce apoptosis [35–37]. When analyzing the above information, one can observe the manifold effect of IL-1*β* on cartilage by inhibiting its restoration possibility, intensifying its deterioration by enzymes and a direct adverse effect on chondrocytes. In the cells of the joint, IL-1*β* is able to induce its own secretion in an autocrine manner to stimulate the synthesis of other cytokines such as, for example, TNF*α*, IL-6, IL-8, and CCL5 chemokine [38–42]. IL-1*β* has been shown to inhibit the signal pathway of the receptor-regulated SMADs (R-SMAD), crucial to the activation of transcription factors associated with TGF-*β* [43]. This is done by increasing the expression of the inhibitor protein SMAD7 and inhibition of synthesis of the TGF-*β* type II receptor in chondrocytes. In addition, effects are observed on the secretion of a number of other enzymes and mediators involved in the pathophysiology of OA. These compounds may include the iNOS generating NO, phospholipase A2 (PLA2), cyclooxygenase-2 (COX-2), prostaglandin E synthase 2 (PGE2 synthase) producing prostaglandin E2 (PGE2) [44–46]. During the course of the disease, IL-1*β* stimulates the production of reactive oxygen species (ROS), which generate the formation of, for example, peroxides and hydroxylated radicals, which directly damage the articular cartilage; the intensification of this process is also associated with decreased expression of oxidative enzymes, which is observed in the joint affected by the disease [47].

### 2.2. Tumor Necrosis Factor Alpha (TNF*α*)

**Figure 3:**
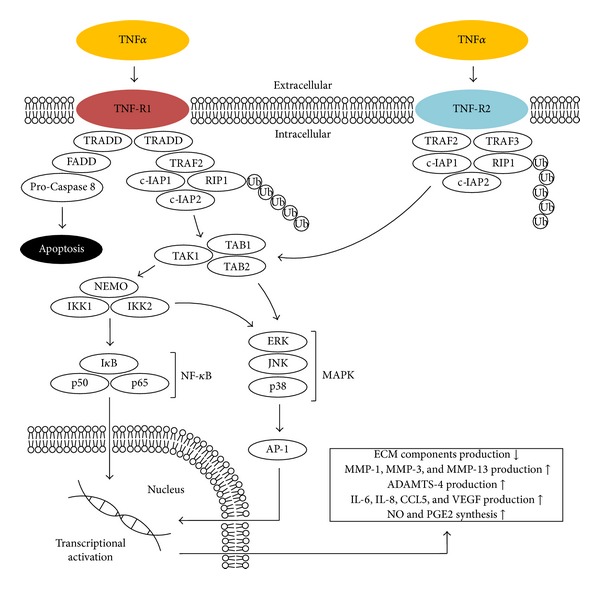
TNF*α* associated intracellular signaling pathways and downstream cellular targets and effects. TNF-R1: tumor necrosis factor receptor superfamily member 1; TNF-R2: tumor necrosis factor receptor superfamily member 2; TRADD: tumor necrosis factor receptor type 1 associated death domain protein; FADD: Fas-associated protein with death domain; TRAF2: TNF receptor-associated factor 6; c-IAP1: also known as Baculoviral IAP repeat-containing protein 2 (BIRC2); c-IAP2: also known as Baculoviral IAP repeat-containing protein 3 (BIRC3); RIP1: receptor-interacting protein kinase 1; Ub: ubiquitin; TRAF3: TNF receptor-associated factor 3; TAK1: also known as mitogen-activated protein kinase kinase kinase 7 (MAP3K7); TAB1: also known as mitogen-activated protein kinase kinase kinase 7 interacting protein 1 (MAP3K7IP1); TAB2: also known as mitogen-activated protein kinase kinase kinase 7 interacting protein 2 (MAP3K7IP2); p50, p65: subunits of proteins forming NF-*κ*B; I*κ*B: (inhibitor of *κ*B) an endogenous complex of proteins inhibiting the activation of NF-*κ*B; IKK1,2/NEMO: NF-*κ*B inhibitor kinase 1,2 (I*κ*B kinase 1,2)/NF-*κ*B kinase inhibitor (NF-*κ*B essential modulator); ERK: extracellular-signal-regulated kinase; JNK: c-Jun N-terminal kinase; p38: p38 mitogen-activated protein kinases; MAPK: mitogen-activated protein kinases; AP-1: activator protein 1.

Tumor necrosis factor alpha (TNF*α*), together with IL-1*β*, is considered a key inflammatory cytokine involved in the pathophysiological processes occurring in the course of OA (Figure 3). It is one of the 19 ligands within the tumour necrosis factors superfamily (TNF superfamily) [48]. It is originally formed as a homotrimeric transmembrane protein type II (mTNF*α*), which can be secreted into the environment in the next stage involving the TACE/ADAM17 metalloproteinase, creating a free form of TNF*α* (sTNF*α*) [49–51]. TNF*α* is secreted by the same cells in the joint that synthesize IL-1*β*, and its increased concentration is also observed in the same elements, such as synovial fluid, synovial membrane, cartilage, and subchondral bone layer, where increased levels of IL-1*β* are also detected [12, 13, 15, 17]. The cytokine has the ability to bind to the two isotypes of membrane receptors located on the surface of almost every nucleated cell TNF-R1 (p55, CD120a, and TNFRSF1a) and TNF-R2 (p75, CD120b, and TNFRSF1b) [52, 53]. The TNF-R1 receptor can be efficiently activated by the soluble and membrane forms, while TNF-R2 mainly binds the membrane form [53]. So far the participation of TNF-R1 seems to have a greater impact on the local loss of articular cartilage than TNF-R2; this, however, does not change the fact that both receptors are involved in signal transduction after being activated by TNF*α* in the processes occurring in OA [54, 55]. The expression of TNF-R1 is also increased within the FLS cells [56, 57]. As mentioned above, both receptors differ in their affinity to the TNF*α* and also in their amino acid composition, degree of glycosylation, and structure, primarily in their intracellular part, which within the TNF-R1 includes the so-called death domain (DD), which does not occur in the TNF-R2 receptor [51, 58]. In view of the significant lack of a homology in the intracellular portion, both receptors have the ability to transmit different signals. TNF-R1 is able to recruit two different TNF-R1 signalling complexes (TNF-RSC) [59, 60]. Complex I is mainly involved in the activation of pathways whose end products stimulate an inflammatory response, especially the production and secretion of cytokines and the production of proteins that prevent apoptosis, whereas the main function of complex II involves signal transduction leading to cell disintegration [60, 61]. The association of TNF*α* with TNF-R1 causes interaction between the TRADD adapter protein with the DD domain and gradual binding of other adapter proteins, such as TRAF2, c-IAP1, c-IAP2, and RIP1 [62–64]. The creation of the complex is followed by ubiquitination of the RIP1 protein, which also binds TAK1, TAB1, and TAB2, resulting in the phosphorylation of the IKK complex and ultimately, the activation of one of the most important transcription pathways in the course of OA—NF-*κ*B [65, 66]. Furthermore, during the formation and effect of complex I, the other important signalling pathway is also activated by JNK kinase, as well as others such as extracellular-regulated kinase (ERK) pathway and p38MAPK [60, 67, 68]. The formation of complex II is accompanied by endocytosis of the activated receptor, changing its conformation and recruitment of FADD and pro-Caspase 8, which ends with cell death [59, 69]. In turn, binding mTNF*α* to the TNF-R2 receptor will result in the binding and mutual interaction of proteins, which includes TRAF2, which is crucial in the signal transduction and others such as TRAF3, c-IAP1, and c-IAP2. The effect of interaction of the complex is the activation of the JNK kinase and the transcription factor NF-*κ*B [70–73]. It has been proven that polymorphism in the gene (M196R) encoding the receptor protein TNF-R2 may predetermine the development of OA by increasing the number of receptor proteins on the surface of chondrocytes, which leads to disturbance of their functions due to excessive activation by mTNF*α* [74]. Moreover, not only polymorphism in the receptor but also in the TNF*α* ligand may favour the occurrence of OA in relation to the specific populations studied [75, 76]. It is worth to mention the presence of an additional ligand capable of incorporating both TNF-R1 and TNF-R2 with still increasing importance in the pathogenesis of OA, which is progranulin (PGRN) also known as granulin epithelin precursor (GEP), PC-cell derived growth factor (PCDGF), proepithelin (PEPI), or acrogranin [77–80]. PGRN is a growth factor having anti-inflammatory and immunomodulatory properties [81]. It was shown that PGRN level is significantly elevated in patients suffering from cartilage arthropathies including OA [82]. The PGRN binding ability to the dedicated TNF*α* receptors, as well as its elevated levels during the course of the disease, makes it a natural antagonist of TNF that interfere with the signaling pathway TNF*α*/TNF-R1 and TNF*α*/TNF-R2 [83]. This implies that an imbalance of TNF*α*/PGRN can both accelerate and inhibit the development of OA. In addition, the presence of PGRN autoantibodies was observed in some joint diseases with an autoimmune component [84–86]. The effect of TNF*α* in most cases coincides with the action of IL-1*β*, and in the case of many phenomena occurring in the course of OA there is a marked synergism between the two cytokines [87]. This effect is the result of activation of the same group of intracellular signalling pathways, which in turn triggers similar effects that increase the inflammation and catabolism in joint tissues [26, 27]. TNF*α* affects blocking the chondrocytes' synthesis of proteoglycan components, proteins binding proteoglycans, and type II collagen [88, 89]. Activated chondrocytes also produce MMP-1, MMP-3, MMP-13, and ADAMTS-4 [33, 90, 91]. As described earlier, there is an induction of chondrocyte death and a disorder in the migration of chondrogenic progenitor cells (CPCs), which strips the cartilage of any possibility of regeneration [35–37, 92]. A clear impact of TNF*α* and IL-1*β* on reducing the efficiency of the respiratory chain was also observed, and hence the decrease in ATP produced within the mitochondria located in chondrocytes; additionally, there is a decrease in the potential of the mitochondrial membrane [93]. TNF*α* is responsible for increased synthesis of, for example, IL-6, IL-8, RANTES, and VEGF [39–41, 94]. Moreover, as described above, IL-1*β* and TNF*α* induce the production of iNOS, COX-2, and PGE2 synthase, thereby increasing the amounts of their products [44, 46, 95, 96].

### 2.3. Interleukin-6 (IL-6)

Interleukin-6 (IL-6) is a compound characterized by omnidirectional interactions in the processes occurring in the human body. It is considered a cytokine that strongly activates the immune system and enhances inflammatory response, although considering some of its effects, it may be classified as anti-inflammatory interaction. IL-6 is a glycoprotein consisting of 184 amino acid residues, which in the process of posttranslational processing ultimately becomes an interconnected structure of four *α*-helices [97]. The production of IL-6 in the tissues of the affected joint is usually in response to IL-1*β* and TNF*α* and is mainly implemented by chondrocytes, osteoblasts, FLS, macrophages, and adipocytes [39, 98–102]. The increased concentration of IL-6 is present in both the synovial fluid and serum and is positively correlated with the intensity of lesions in X-ray imaging [103–107]. The effect of IL-6 can be observed through a unique receptor system. There are two subtypes of the IL-6R receptor (gp80, CD126), namely, the membrane form of mIL-6R and the soluble sIL-6R, which may be formed by cutting or by the ADAM17/TACE by metalloproteinase or by means of alternative splicing [108]. Transferring the signal into the cell requires the association of additional gp130 protein (CD130) [109, 110]. The gp130 protein is also present in the membrane form mgp130 and in soluble form sgp130 [111, 112]. The association of the IL-6 and IL-6R with sgp130 is associated with inhibiting the signalling pathway of IL-6 [111, 112]. Recruitment of the ligand-receptor complex to mgp130 causes homodimerization and consequently the formation of hexamer that allows effective signal transduction to the cell [113]. JAK kinase mediates the phosphorylation of tyrosine residues that are an integral part of the gp130, which in further stages results in the activation of STAT3, phosphorylation of MAPK, and activation of the PI3 K/AKT pathway [114, 115]. While analyzing the role of IL-6 in OA, one should first note that the polymorphism of gene (−174G/C) encoding IL-6 may predetermine the development of OA [116, 117]. The effect of IL-6 on joint cartilage is not different from other cytokines and, in synergy with them, causes a decrease in the production of type II collagen and increases the production of enzymes from the MMPs group [118–120]. It was also found that these effects can be enhanced under the influence of injury [121]. IL-6 is considered to be the key cytokine, which causes changes in the subchondral bone layer [122, 123]. Its effect is largely based on promoting the formation of osteoclasts and thus bone resorption while showing synergism with IL-1*β* and TNF*α* [122, 124]. Osteoblasts stimulated by IL-1*β*, TNF*α*, and IL-6 become a source thereof and may also produce MMPs by adversely affecting the cartilage located near it [124]. In addition to the demonstrated role of other cytokines in inducing the production of IL-6, its secretion by osteoblasts, chondrocytes is also affected by prostaglandin E2 [125, 126]. In turn, while analyzing tests on animal models, it can be observed that IL-6 may have a different effect in some situations. An experiment performed on mice lacking the gene for IL-6 showed that they exhibited a tendency to develop much more advanced degenerative changes than healthy mice [127]. During another experiment, it was also demonstrated in mice lacking the gene for IL-6 that intra-articular injection of IL-6 reduces the loss of proteoglycans in the acute phase of chronic joint inflammation and induces the formation of osteophytes [128].

### 2.4. Interleukin-15 (IL-15)

Interleukin-15 (IL-15) is a glycoprotein that takes the form of four interconnected *α*-helices with a mass of 14-15 kDa [129, 130]. Its action is mainly based on the stimulation of differentiation and proliferation of T cells and NK cells [131]. It is one of the better documented cytokines involved in the pathogenesis of rheumatoid arthritis (RA) [132, 133]. While analyzing the involvement of IL-15 in the pathogenesis of OA, its increased concentration was found in the synovial fluid in the early stages of the disease [134]. It has also been shown that the increased IL-15 level in the serum correlates with both the sensation of pain and the severity of lesions in the X-ray image [135]. It has also been noted that its presence can stimulate the secretion of certain types of metalloproteinases from the MMPs group [134].

### 2.5. Interleukin-17 (IL-17)

The family of interleukins-17 (IL-17) is a group of cytokines with inflammatory effect, which draws more and more attention of researchers for its participation in the pathogenesis of OA. It consists of six members (IL-17A-F) that can interact through five types of receptors (IL-17RA-E) [136, 137]. The source of IL-17 is mainly stimulated CD4^+^ T cells and mast cells that infiltrate the synovial membrane and the entire joint through blood vessels [138–141]. The main cells in the joint that are affected by IL-17 are chondrocytes and FLS exhibiting the expression of IL-17R on their surface [142]. In addition, studies have demonstrated direct cellular immune response of those T cells against membrane antigens of chondrocytes and fibroblasts in the course of OA [143]. The level of IL-17 measured in the serum and the synovial fluid of patients is elevated and shows a positive correlation with the radiographic image of lesions in OA [144]. IL-17 has been shown to inhibit the synthesis of proteoglycans by chondrocytes and promotes the production of enzymes of the MMPs group [145–147]. Furthermore, IL-17 influences the secretion of other cytokines and compounds negatively affecting the cartilage, such as IL-1*β*, TNF*α*, IL-6, NO, and PGE2 [148–150]. The effect of IL-17 on the secretion of VEGF by both chondrocytes and FLS is also characteristic; it favours the excessive development of blood vessel network within the synovial membrane, leading to its hypertrophy [94, 142]. It was also confirmed recently that the polymorphism the gene IL-17A G-197A in certain populations may correlate with the susceptibility to the development of OA [151].

### 2.6. Interleukin-18 (IL-18)

Interleukin-18 (IL-18) is another representative of the IL-1F family [9]. It is generated as a precursor form of the pro-IL-18, consisting of 192 amino acid residues, which is transformed into a biologically active form after the activation of Caspase 1 or proteinase 3, comprising 157 amino acid residues [152, 153]. It has been noted that the level of Caspase 1 is elevated both in the articular cartilage and synovium in OA patients which greatly promotes the formation of IL-1*β* and IL-18 [154]. The production of IL-18 in the joint is determined by chondrocytes, osteoblasts, FLS, and macrophages [155–157]. Its increased concentration is evident in the synovial fluid, synovium, cartilage, and blood serum and shows a positive correlation with the degree of severity of the disease seen in radiographic images [158–160]. The effect of IL-18 is mediated by the IL-18R*α* receptor, which bears structural and functional similarity to IL-1R. As in the case of IL-1*β*, efficient signal transmission by IL-18 requires not only the presence of IL-18R*α* but also the additional recruitment of the IL-18R*β* (IL-18RAcP) chain [161, 162]. Intraventricular signal transmission through a heterodimeric receptor complex IL-18 does not show significant differences from the mechanism of signal transduction by the IL-1*β* receptor complex [163]. Studies have shown that polymorphism of genes encoding both IL-18R and the IL-18 interleukin in some variants may predetermine both the development of OA and lumbar disc degeneration (LDD) [164, 165]. IL-18 affects chondrocytes by inducing the upregulation of IL-18R*α* on their surface and stimulation excess synthesis of metalloproteinases MMP-1, MMP-3, and MMP-13 [166]. In addition to increasing the concentration of cartilage degrading enzymes, there is an inhibition of production of proteoglycans, aggrecan, and type II collagen; moreover, chondrocytes exhibit morphological changes typical of cells entering apoptosis [167–169]. IL-18 affects chondrocytes and synovial cells, increasing the production of a range of compounds and enzymes, such as the IL-18 in an autocrine manner, IL-6, iNOS, PGE2, COX-2, and VEGF [155, 170–173].

## 3. Anti-Inflammatory Cytokines

**Figure 4:**
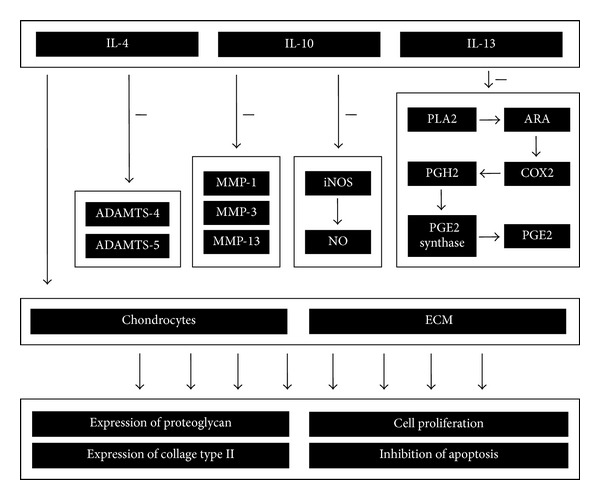
Schematic of the anti-inflammatory and chondroprotective effect of IL-4, IL-10, and IL-13 on articular cartilage during the course of OA.

The main representatives of the group of anti-inflammatory cytokines involved in the pathogenesis of OA are IL-4, IL-10, and IL-13. In this chapter, the authors focus on presenting the effect of only those of the above cytokines, whose biological activity is the most important in the light of the relevant problems (Figure 4). However, it should be noted that IL-4, IL-10, and IL-13 belong to a broader group of anti-inflammatory cytokines and are not the only representatives.

### 3.1. Interleukin-4 (IL-4)

Interleukin-4 (IL-4) is a protein composed of 129 amino acids, which takes the form of four interconnected *α*-helices additionally stabilized by three disulfide bonds [174–176]. IL-4 is a ligand whose biological activity is mediated through a receptor system dedicated to both IL-4 and IL-13 [177]. There are two types of receptors forming heterodimers which are structurally a combination of two or three receptor chains, such as IL-4R*α*, IL-13R*α*1, and IL-2R*γ*c [178]. The dimerization of IL-4R*α* and IL-2R*γ*c (type 1 complex) enables the attachment of IL-4, while the interaction between IL-4R*α* and IL-13R*α*1 (type 2 complex) enables efficient attachment of both IL-4 and IL-13 [177–179].

After the formation of the receptor type 1 complex, an intracellular signal transduction takes place by gradual phosphorylation of the IL-4R*α*/JAK1/STAT3/STAT6 cascade, leading to the expression of several proinflammatory genes [180, 181]. The observed polymorphism of the genes encoding both IL-4 and IL-4R*α* may predetermine the development of OA in the joints of the hand, knee and hip joint gives reason for claiming that the interaction between IL-4 and its dedicated receptor complex is one of the elements of the pathogenesis of the disease [182–184]. The production of IL-4 is primarily determined by T cells (Th2) infiltrating the synovium of the joint by blood vessels [185, 186]. It was further found that the level of soluble IL-4R*α* (sIL-4R) is elevated in the serum of patients suffering from OA as compared to the healthy control group [187]. The increased concentration of IL-4 is also observed in the synovial fluid and synovial cells [185, 188, 189]. IL-4 is associated with a strong chondoprotective effect. In a number of studies it was found that IL-4 has an inhibiting effect on the degradation of proteoglycans in the articular cartilage, by inhibiting the secretion of MMPs metalloproteinases, as well as reducing the variation in the production of proteoglycans that are visible in the course of OA [190–193]. Interestingly, in the course of OA, chondrocytes showed decreased susceptibility to the effects of IL-4 which may be responsible for the rapid degeneration of the articular cartilage [194–196]. In addition, IL-4 alone or in combination with IL-10 exhibits properties inhibiting the apoptosis of both the chondrocytes and FLS [191, 197, 198]. Considering the effect of IL-4 on cell cultures of chondrocytes and FLS treated with it, there is a decrease of synthesis of inflammatory cytokines such as IL-1*β*, TNF*α*, and IL-6 [191, 197, 199]. Simultaneously, IL-4 can induce upregulation of the expression of TNF*α* receptors, such as TNF-R1, and TNF-R2 [200]. In addition to a direct decrease in the secretion of inflammatory cytokines, there is also a decrease in the secretion of other inflammatory mediators such as PGE2, COX-2, PLA2, and iNOS [191, 197, 200, 201]. It seems reasonable to cite a study, which analyzed the effect of rmIL-4 (recombinant mouse IL-4) in combination with rmGM-CSF for the dendritic cells CD11b^+^F4/80^+^iDC in a mouse model, where inflammatory joint disease was induced artificially [202]. The observed results seem to confirm the positive effect of IL-4 and GM-CSF on restoring the balance between the secretion of proinflammatory and anti-inflammatory factors in the immune cells in mice with CIA (collagen-induced arthritis). CD11b^+^F4/80^+^iDC cells exposure to said effect showed an increased ability to synthesize the anti-inflammatory IL-10 and TGF-*β* as well as an inhibitory or excitatory effect relative to other cells of the immune system. It has been noted that proliferation of Th17 lymphocytes has been reduced and that they showed the ability to synthesize and secrete proinflammatory IL-17. Macrophages showed a reduced ability to produce and secrete IL-1 and TNF*α*. In turn, the regulatory T cells were stimulated to increase the production of IL-10 and TGF-*β*. The test mice showed reduced morphological markers of inflammation of the synovial membrane and articular cartilage and decreased immunological activity of the thymus, relative to the control group (unexposed to rmIL-4 rmGM-CSF). Although the mouse model with CIA corresponds mainly to RA in the world literature, the dependencies between the effect of IL-4 and GM-CSF observed for cells of the immune system may be a reference to some of the processes occurring in OA. In the context of other studies, this gives us a rough idea about the anti-inflammatory effect of IL-4 in the course of OA, although it certainly requires further study.

### 3.2. Interleukin-10 (IL-10)

Interleukin-10 (IL-10) is a cytokine structurally related to interferons, which is in the form of a homodimer wherein every monomer is a polypeptide chain consisting of 160 amino acid residues, taking the structure of 6 interconnected *α*-helices [203–205]. Initiation of IL-10 is made possible by binding it to the receptor IL-10R, which is a heterodimer composed of subunits IL-10R1 and IL-10R2 [206, 207]. The first step of activation of the receptor involves binding IL-10 to the IL-10R1 subunit, which then changes its conformation in such a way as to allow binding the IL-10 and IL-10R1 complex to the IL-10R2 subunit [206, 207]. At this point a cascade of intracellular signal transmission is started. This leads to the activation of the tyrosine kinase JAK1 (associated with the IL-10R1 subunit) and TYK2 (associated with IL-10R2) [208]. This in turn implies sending the cellular signal further by the transcriptional activators STAT3, which upon binding to SBE (STAT-binding elements) of the cell nucleus stimulate the synthesis of the products of genes dependent on IL-10 [209]. IL-10 is another cytokine that shows chondoprotective effect in the course of OA. Chondrocytes express both the cytokine IL-10 and the receptor IL-10R [210]. It has been proven that IL-10 is involved in stimulating the synthesis of type II collagen and aggrecan. Following the administration of IL-10 in vitro conditions, both healthy articular cartilage and one in the course of OA demonstrated an increase in proteoglycan synthesis and its percentage share in the extracellular matrix. Furthermore, the synovium of patients with osteoarthritis in the context of hemophilia, which showed a tendency to excessive production of TNF*α* and IL-1*β* following the in vitro administration of IL-10 significantly reduced the average secretion of the mentioned proinflammatory cytokines by 60% and 83%, respectively [211]. It has been shown that IL-10 is responsible for inhibiting the production of MMPs family of metalloproteinases [212]. It was confirmed that IL-10 (similar to IL-4) inhibits the apoptosis of chondrocytes [213]. These properties of IL-10 are likely the result of stimulation of the synthesis of IL-1*β* antagonist, which is IL-1Ra and the tissue inhibitor of metalloproteinases-1 (TIMP-1) as well as growth factors [214, 215]. The study of the effect of IL-10 on the stimulation of chondrocyte proliferation in a mouse model shows further signaling pathways of IL-10 [216]. It has been proved that IL-10 activates the kinase pathway SMAD1/SMAD5/SMAD8 and ERK1/2 MAP and induces the expression of bone morphogenetic proteins 2 and 6 (BMP-2, BMP-6). BMP proteins belong to the TGF-*β* family and play a crucial role in chondrogenesis [217]. They are part of the signaling pathway that affects many genes and proteins responsible for the regulation of the mesenchymal cell transformation in chondrocytes, such as NKX-3.2/SOX9, SOX5, and SOX6 [218, 219]. In addition to determining the transformation of multipotent cells towards chondrocytes, BMP proteins stimulate their proliferation both directly and through the IHH/PTHrP-dependent pathway [220, 221]. The involvement of IL-10 in the induction of differentiation and proliferation of chondrocytes through the BMP signaling pathway was thus confirmed. The study authors deduce that the observed mechanisms may be partly responsible for the chondoprotective effects of IL-10 in OA. In another study, the cells of the haematopoietic system were observed after stimulation by LPS. The aim was to figure out how LPS stimulation affects the synthesis and level increase of IL-10 in blood samples from patients with OA and healthy subjects. Based on the results of IL-10 levels and information on the health status of the subjects, a specific prediction model of OA was developed. Persons whose blood samples showed no significant increase in the level of IL-10 relative to the starting point (the lowest quartile of results) were running even a 3-fold risk of developing OA compared to those whose levels of IL-10 after stimulation were in the highest quartile [222]. IL-10 reduces the effect of TNF*α* on synovial fibroblasts sampled from patients with OA [200]. This is manifest in a significant decrease in their secretion of PGE2, COX-2, and PLA2. Interestingly, IL-10 does not have those properties with respect to the fibroblasts not treated with TNF*α*. It was confirmed that IL-10 reduces the expression of TNF*α* receptors and thus their ability to bind to the surface of fibroblasts. The anti-inflammatory and chondroprotective nature of IL-10 is composed of results of interesting studies of the influence of exercise on the secretion of cytokines in joint fluid and periarticular tissues in patients with OA of the knee joints [223, 224]. In these studies, patients were divided into a study group, which has exercised knee joints affected by OA for 3 h and a control group, which had to refrain from exercise during the same time. It is worth noting that in the control group there were patients with the same degree of severity of OA of the knee joints. Knee joints affected by OA were connected to catheters in both the study and control groups. Catheters, placed into the joints and periarticularly, were used to examine the levels of IL-10 and other cytokines before exercise, during exercise, and after exercise relative to the control group. A significant increase in the level of IL-10 in the synovial fluid and periarticular tissues was observed in patients in the study group as compared to the control group, in which the levels of IL-10 observed before and after exercise showed no significant difference. On the basis of these observations, it seems reasonable to advocate the medicinal effect of physical exercise on joints affected by OA. In truth, it is not clear what mechanism leads to an increased secretion of IL-10. Some authors argue that this is due to the inductive effect of increased intra-articular pressure on cellular secretion [225, 226].

### 3.3. Interleukin-13 (IL-13)

Interleukin-13 (IL-13) is a compound that takes the structure of four interconnected *α*-helices, which is very similar in its effect to IL-4 [227, 228]. Similar to IL-4, the action of IL-13 as a ligand is mediated through a receptor system that combines both cytokines [177]. In this case it requires the recruitment of a ligand, namely, IL-13, and in addition the chains IL-4R*α* and IL-13R*α*1 to create a type 2 complex [177, 178]. With this combination, IL-13 has the capacity to transfer the intracellular signal both by the cascade IL-4R*α*/JAK2/STAT3 and IL-13R*α*1/TYK2/STAT1/STAT6 [180]. The anti-inflammatory and chondoprotective effects of IL-13 on the cells of the immune response, articular cartilage, and synovium in OA have been fairly well documented [229, 230]. The anti-inflammatory effect of IL-13 in the context of OA seems most important with respect to fibroblasts included in the synovium. Note, however, that the anti-inflammatory properties based on the inhibition of the secretion of inflammatory cytokines relate to a whole range of cells such as macrophages, monocytes, B cells, NK cells, and endothelial cells [231]. In one of the most important studies into the effects of IL-13 on OA, synovium samples were taken from 16 patients with OA during the knee endoprosthesis implantation procedure (TKR) [232]. Patients were diagnosed based on the criteria of the ACR Diagnostic Subcommittee for OA, revealing stage III or IV of the disease according to radiological imaging [233]. The synovial samples taken were administered proinflammatory LPS (control samples), followed by IL-13 (test samples). After 72 h incubation, laboratory tests were performed including binding tests, northern blotting, and ELISA. It has been shown that, compared to the control samples, IL-13 showed inhibitory effects on the synthesis of proinflammatory IL-1*β*, TNF*α*, and MMP-3 with a simultaneous increase in the level of IL-1Ra. In the studies synovium cells, the amount of mRNA for IL-1*β* was reduced, while the mRNA level for IL-1Ra was increased relative to control samples. Moreover, synovial fibroblasts showed a reduction in binding between IL-1*β* and its receptor, which was caused by the increased production and action of IL-1Ra. It has been demonstrated that IL-13 has the ability to inhibit the proinflammatory effect of TNF*α* relative to fibroblasts from patients with OA [190]. A significant decrease in PGE2 synthesis by blocking the synthesis of COX-2 was observed. Interestingly, compared to IL-4 and IL-10, IL-13 does not affect the production level of PLA2. This may indicate the selective effect of IL-13 that only involves COX-2 and, more specifically, the expression of its gene. The study proved that IL-13 has the ability to decrease the nuclear concentration of transcription factors C/EBP, which may affect the inhibition of the synthesis of COX-2. In this context, fibroblasts may exhibit properties similar to osteoblasts [234]. The analysis of these results indicates the potential utility of IL-13 in the treatment of OA, as a compound that inhibits the inflammatory processes, protects chondrocytes, reduces the secretion of inflammatory cytokines and metalloproteinases, while stimulating the synthesis of IL-1Ra.

## 4. Summary

OA is a set of complex and difficult-to-identify processes leading to progressive degeneration of joints. But, contrary to what it may seem to be, it is not only a set of catabolic effects. Apart from them, anabolic anti-inflammatory processes also occur continually. Evident imbalance in metabolism observed in OA is the final manifestation of the dysregulation within the so-called cytokine network between anti-inflammatory and inflammatory cytokines. In this paper, current knowledge on the impact of the most important cytokines in the pathogenesis of OA has been presented, based on world literature survey. Cytokines were categorized with respect to their biological effect as inflammatory and anti-inflammatory. However, this criterion is not so obvious in particular conditions. Respective cytokines should be analysed together with the nature and structure of specific receptors and the intracellular signalling pathways triggered by their activation in the specific tissue. Thus, particular attention was paid to the potential impact of cytokines on articular cartilage, synovium and the cells of the immune system. In the context of developing optimal treatment tactics for OA, the authors of this paper have tried to summarize and comment on the most important information resulting from the collected above (published) data.

Due to their effects within the joint, inflammatory cytokines have a primarily destructive impact on articular cartilage. It is a multilevel impact that involves not only the induction of aging and apoptosis of chondrocytes, but also a decrease in the synthesis of the key components of ECM, such as proteoglycans, aggrecan, and type II collagen. In addition, inflammatory cytokines contribute to the increased synthesis and release of many proteolytic enzymes that decompose articular cartilage, which include the metalloproteinases family MMPs and ADAMTS. Apart from their effect on chondrocytes, synovial cells and other articular, and periarticular tissues, inflammatory cytokines affect the cells of the immune system migrating to the site of inflammation. Due to the action of inflammatory cytokines, all of these cells tend to produce excessive amounts of inflammatory PGE2, COX-2, phospholipase A2, NO, and free radicals. It is worth noting that inflammatory cytokines stimulate cells to synthesize other inflammatory cytokines, namely, IL-1*β*, TNF*α*, IL-6, IL-8, and chemokines CCL5 (RANTES). They can thereby also promote their own production, thus demonstrating an autocrine, paracrine, and self-propelling effect on the inflammation process.

The action of anti-inflammatory cytokines mainly involves inhibiting the synthesis of inflammatory cytokines, particularly IL-1*β* and TNF*α*. Observed effects of anti-inflammatory cytokines include increased proteoglycan synthesis, inhibited apoptosis of chondrocytes, decreased synthesis and secretion of metalloproteinases, and decreased level of PGE2. However, these properties should be treated as an inhibitory effect on inflammatory cytokines rather than direct chondoprotective capabilities. It should be added that it has been proven that the action of anti-inflammatory cytokines is mainly performed relating to cells stimulated by inflammatory cytokines, whereas no significant differences are noted in the metabolism of cells not subject to such stimulation. Such a relationship has been observed, for example, regarding synovial fibroblasts obtained from patients with OA [200]. However, certain properties of anti-inflammatory cytokines go beyond the simplest idea of antagonism with respect to inflammatory cytokines. For example, IL-10 stimulates the synthesis of growth factors of the BMP family (BMP-2, BMP-6) and affects the regulatory proteins such as NKX-3.2/SOX9, SOX5, and SOX6 which induces and regulates the process of chondrogenesis. In turn, rmIL-4 in a mouse model stimulates regulatory T cells to synthesize IL-10 and the growth factor TGF-*β*, which at least corresponds to the properties of inflammatory cytokines in the context of stimulating their own synthesis [202]. The dominant observation concerning the effects of inflammatory and anti-inflammatory cytokines in the course of OA is that the inflammatory response within the affected joints is never controlled completely. Many authors have confirmed that the effect of anti-inflammatory cytokines and the treatment based on them cannot positively stop the progression of the disease by blocking the promotion of catabolic pathways [200]. So far, there have been no comprehensive answers to this issue. According to the standpoint of the authors of this study, the insufficient effect of anti-inflammatory cytokines in the joint may be determined by the characteristics of their action. Relative to healthy joint tissue, anti-inflammatory cytokines show no significant anabolic effect likely to create a sufficient biochemical advantage against the effects of inflammatory cytokines. It is only in a joint affected by the disease in the presence of inflammatory mediators that the role of anti-inflammatory cytokines becomes noticeable. The effect of anti-inflammatory cytokines therefore shows no primary prophylactic capability; however, it has the properties of a secondary response to combat the symptoms of the disease. Such blocking effect of the activity of inflammatory cytokines on the “action-reaction” principle, already in the early stages of the disease places the anticatabolic processes as secondary in relation to the catabolic effect and is therefore late or delayed.

In search of a better understanding of the pathomechanism of OA, one should pay attention to the quantitative changes that occur in the so-called cytokine network. The abundance or deficiency of any element can cause disturbances of the whole structure and ultimately its collapse. In OA, there is a lot of missing “building blocks” that contribute to preservation of the metabolic balance in the joint. The main figure in this group is primarily TGF-*β*, a cytokine and growth factor responsible for stimulating the production of proteoglycan, type II collagen, and chondrogenesis [235, 236]. Its presence in the joints is observed in healthy subjects, as opposed to patients with OA, where the amounts of TGF-*β* are low or even undetectable [237]. Some authors believe that reducing the level of this cytokine is responsible for sensitizing the cartilage to the effect of inflammation mediators and for the progress of the disease [235]. The explanation for the reduced amount of TGF-*β* in the course of OA and thus also its biological activity may involve intercepting the cell signalling pathways by inflammatory cytokines, although this certainly requires further investigation [238, 239]. Another factor, which may contribute to the unrestrained progression of OA, is the cell type responsible for the production of specific cytokines. Namely, in the OA model, the production of anti-inflammatory cytokines is determined primarily by cells of the immune system, which are largely infiltrating cells. In turn, inflammatory cytokines such as IL-1*β* or TNF*α* are synthesized and secreted by the cells of the immune system, as well as locally by articular cartilage cells [240]. This dual model of release seems to create favourable conditions for the development of OA. Add a slightly visible influence of anti-inflammatory cytokines on stimulating their own secretion and thus the lack of a “self-supplying” mechanism (as opposed to inflammatory cytokines), and we will see the image of the disease, in which it is not possible to bring about the advantages of inflammatory processes. Such a scheme of the progress disease quite accurately corresponds to the image of OA progression. Currently we are trying to seek appropriate treatment likely to stop the progress of OA or at least slow it down to a satisfactory degree. The tactics used by researchers are to influence cell transduction pathways that are crucial to the induction of inflammatory process. Therefore, research is underway, aimed at obtaining more accurate knowledge of the effect of inflammatory and anti-inflammatory cytokines. The emerging picture shows the multiplicity, complexity, and the multilevel nature of processes taking place with the participation of cytokines in the course of OA. Their discovery and understanding is a big challenge for today's medicine; however, it seems absolutely crucial to finally develop effective methods of treatment.
